# OXA-48 Carbapenemase-Encoding Transferable Plasmids of *Klebsiella pneumoniae* Recovered from Egyptian Patients Suffering from Complicated Urinary Tract Infections

**DOI:** 10.3390/biology10090889

**Published:** 2021-09-09

**Authors:** Ann A. Elshamy, Sarra E. Saleh, Mohammad Y. Alshahrani, Khaled M. Aboshanab, Mohammad M. Aboulwafa, Nadia A. Hassouna

**Affiliations:** 1 Department of Microbiology and Immunology, Faculty of Pharmacy, Ain Shams University, Organization of African Unity St., P.O. Box 11566, Cairo 11566, Egypt; ann.elshamy@pharm.asu.edu.eg (A.A.E.); sarradeif@pharma.asu.edu.eg (S.E.S.); or mohammad.aboulwafa@ksiu.edu.eg (M.M.A.); nadia.hassouna@pharma.asu.edu.eg (N.A.H.); 2 Department of Clinical Laboratory Sciences, College of Applied Medical Sciences, King Khalid University, P.O. Box 61413, Abha 9088, Saudi Arabia; moyahya@kku.edu.sa; 3 Faculty of Pharmacy, King Salman International University, Ras-Sedr 46612, Egypt

**Keywords:** antimicrobial resistance, carbapenemase genes, urinary tract infections, plasmids, Gram-negative bacteria, carbapenem-resistant *Enterobacteriaceae*

## Abstract

**Simple Summary:**

Gram-negative bacteria are common causes of urinary tract infections (UTIs), some of which can resist treatment by antibiotics, including carbapenems, which are last resort treatment options. This study aimed to report the resistance of some Gram-negative bacteria causing complicated UTIs to carbapenems at two important hospitals in Cairo, Egypt, and to determine the possible transfer of this resistance to other bacterial species. The collected bacteria were tested for antibiotic resistance and detection of the genes responsible for this resistance. A total of 256 Gram-negative bacterial clinical isolates were collected, 65 (25.4%) of which showed carbapenem resistance. The detected carbapenem resistance genes were *bla*_OXA-48_, *bla*_VIM_, *bla*_KPC_, and *bla*_NDM_ genes. The *bla*_OXA-48_, among other genes, was successfully transferred to a previously susceptible bacteria, making it resistant. The study concluded that the rate of carbapenem resistance among Gram-negative bacteria causing UTIs in Cairo, Egypt is relatively high and can be transferred among bacterial hosts.

**Abstract:**

Gram-negative bacteria are common causes of urinary tract infections (UTIs). Such pathogens can acquire genes encoding multiple mechanisms of antimicrobial resistance, including carbapenem resistance. The aim of this study was to detect the carbapenemase-producing ability of some Gram-negative bacterial isolates from urine specimens of patients suffering from complicated UTIs at two vital tertiary care hospitals in Cairo, Egypt; to determine the prevalence of carbapenemase genes among plasmid-bearing isolates; and explore the possibility of horizontal gene transfer to other bacterial species. The collected isolates were subjected to antimicrobial susceptibility testing, phenotypic analysis of carbapenemase production, and molecular detection of plasmid-borne carbapenemase genes, then the extracted plasmids were transformed into competent *E. coli* DH5α. A total of 256 Gram-negative bacterial clinical isolates were collected, 65 (25.4%) isolates showed carbapenem resistance of which 36 (55.4%) were carbapenemase-producers, and of these 31 (47.7%) harbored plasmids. The extracted plasmids were used as templates for PCR amplification of *bla*_KPC_, *bla*_NDM_, *bla*_VIM_, *bla*_OXA-48,_ and *bla*_IMP_ carbapenemase genes. The *bla*_OXA-48_ gene was detected in 24 (77.4%) of the tested isolates while *bla*_VIM_ gene was detected in 8 (25.8%), both *bla*_KPC_ and *bla*_NDM_ genes were co-present in 1 (3.2%) isolate. Plasmids carrying the *bla*_OXA-48_ gene from 4 *K. pneumoniae* clinical isolates were successfully transformed into competent *E. coli* DH5α. The transformants were carbapenemase-producers and acquired resistance to some of the tested antimicrobial agents as compared to untransformed *E. coli* DH5α. The study concluded that the rate of carbapenem resistance among Gram-negative bacterial uropathogens in Cairo, Egypt is relatively high and can be transferred horizontally to other bacterial host(s).

## 1. Introduction

Urinary tract infections (UTIs) represent one of the most widespread bacterial infections that require antimicrobial treatment [1,2]. They are caused by uropathogens that are capable of colonization in the naturally sterile urinary tract [3]. UTIs can either be uncomplicated or complicated, the difference being that uncomplicated UTIs affect individuals that have no functional or anatomical abnormalities of the urinary tract, while complicated UTIs (cUTIs) are associated with anatomically abnormal urinary tracts and/or the presence of one or more risk factors complicating the infection. These risk factors include urolithiasis, renal insufficiency, urinary obstruction, draining devices and indwelling catheters, voiding dysfunction, surgery of the urinary tract, compromised immune system, diabetes mellitus, the male gender, and pregnancy [4,5]. Antimicrobial resistance and subsequent treatment failure are common in cUTI [6].

The emergence of antimicrobial resistance by bacteria which produce extended spectrum β-lactamase enzymes (ESBLs) has limited the effectiveness of β-lactams used to treat UTIs [7]. In addition to this, co-resistance among uropathogens to β-lactams, aminoglycosides, and fluoroquinolones further limits the available treatment options [8]. As ESBL-producers spread throughout healthcare facilities, carbapenems such as imipenem/cilastatin, meropenem, doripenem, and ertapenem were used more frequently, and were regarded as the last resort treatment for infections caused by ESBL-producers and multidrug-resistant (MDR) pathogens [9,10]. Unfortunately, the increased use of carbapenems has led to the emergence of carbapenem resistance (CR) in Gram-negative bacteria (GNB), such as *Enterobacterales*, *Pseudomonas* spp., and *Acinetobacter* spp., as well as the emergence of pathogens carrying up to three different carbapenemase genes. These CR pathogens are capable of spreading in the hospital setting and in the community [11,12]. The rapid spread of CR has posed a global public health crisis owing to the lack of novel antimicrobials that could be used as an alternative last resort treatment [13,14].

There are three major mechanisms of CR: overexpression of efflux pumps, porin-mediated resistance, and enzyme-mediated resistance [15,16]. The latter is due to the production of carbapenemases, which are β-lactamase enzymes capable of hydrolyzing β-lactam antimicrobials and carbapenems. Based on the Ambler classification system, carbapenemases belong to three classes of β-lactamases according to their chemical structures and substrate specificity: classes A, B, and D [15]. Classes A and D possess a serine residue at the active site that facilitates β-lactam ring opening; they are therefore called serine β-lactamases (SBLs) [17]. Class B comprises metallo-β-lactamases (MBLs), the active site of which contains zinc ions, hence their name [18].

Most carbapenemase-producers (CPs) are MDR pathogens carrying multiple resistance determinants to other antimicrobial agents [19]. Carbapenemase-mediated resistance is of great concern, as these enzymes are encoded by plasmid-borne genes which can be transferred horizontally to other bacterial species [10]. The most common carbapenemases, based on carbapenem hydrolysis and geographical dissemination of outbreaks, are *Klebsiella pneumoniae* carbapenemases (KPC), New Delhi metallo-β-lactamase (NDM), imipenem-resistant *Pseudomonas*-type carbapenemases (IMP), Verona integron-encoded metallo-β-lactamase (VIM), and oxacillinase (OXA-48-like) types. They are encoded by *bla*_KPC_, *bla*_NDM_, *bla*_IMP_, *bla*_VIM_, and *bla*_OXA-48_ genes, respectively [20,21]. *Enterobacterales* are extensively reported as CPs, especially *Klebsiella pneumoniae* and *Escherichia coli*. However, carbapenemase genes and their mobile genetic elements (MGEs) are not limited to these species [22]. CR, especially due to carbapenemase production, is already widespread and well-reported in some parts of the world including Europe, South America, and Asia, while the situation is not well-reported in other parts such as Africa [14].

Our aim was to detect the carbapenemase-producing ability of GNB isolates recovered from urine specimens of patients suffering from cUTIs at two vital tertiary care hospitals in Cairo, Egypt, to detect the prevalence of carbapenemase genes among plasmid-bearing isolates, and to explore the possibility of horizontal gene transfer to other bacterial host(s).

## 2. Materials and Methods

### 2.1. Collection of Clinical Isolates

Starting November 2019 to November 2020, 256 Gram-negative clinical bacterial isolates were collected from the microbiology laboratories of El-Demerdash and Kasr Al-Ainy Tertiary Care Hospitals (each with approximately 3200 beds), Cairo, Egypt. According to the hospitals’ records, all isolates were non-duplicates and were recovered from urine specimens of patients suffering from cUTIs. Written and oral informed consents were obtained from the patients or their legal guardians after explaining the purpose of the study. The inclusion criteria were: admission to the above-mentioned hospitals during the study period; patients having colony count of uropathogens >10^5^ cfu/mL in urine sample, indicating significant bacteriuria; patients with risk factors of cUTIs (urolithiasis, renal insufficiency, urinary obstruction, indwelling catheters, voiding dysfunction, surgery of the urinary tract, diabetes mellitus); and consent of the patient or legal guardian. Patients with uncomplicated UTIs were excluded from the study. The study complied with the principles laid out in the Declaration of Helsinki and was approved by the ethics committee of Faculty of Pharmacy Ain Shams University (ENREC-ASU-2019-98).

The identification of isolates was based on their microscopic, morphologic, and biochemical characteristics as stated in Bergey’s manual of determinative bacteriology [23]. The bacterial identification was confirmed by comparing the results to the hospitals’ records. Identification of carbapenem-resistant isolates was further confirmed by using the commercially available API^®^ 20E identification kit (BioMérieux^®^ SA, Marcy l’Etoile, France).

### 2.2. Antimicrobial Susceptibility of the Collected Isolates

The Kirby–Bauer disk diffusion test was performed on all collected isolates [24] using 4 carbapenem disks including imipenem (10 μg), meropenem (10 μg), ertapenem (10 μg), and doripenem (10 μg), followed by measuring the inhibition zone diameters, then interpreting the susceptibility by referring to Clinical and Laboratory Standards Institute (CLSI) guidelines 2020 [25]. Isolates that showed resistance to at least one of the above-mentioned carbapenems were considered CR-GNB, and were selected for further studying. They were each tested against a panel of 13 additional disks of antimicrobials commonly used to treat UTIs, including amoxicillin/clavulanic acid (30 μg), ampicillin/sulbactam (20 μg), cefoxitin (30 μg), ceftazidime (30 μg), ceftriaxone (30 μg), cefepime (30 μg), amikacin (30 μg), gentamicin (10 μg), ciprofloxacin (5 μg), levofloxacin (5 μg), trimethoprim/sulfamethoxazole (25 μg), nitrofurantoin (300 μg), and fosfomycin (200 μg). The reference strain *E. coli* ATCC^®^ 25922 was used as control.

### 2.3. Phenotypic Detection of CPs

#### 2.3.1. Blue-Carba Test

This test was validated for the detection of CPs directly from bacterial cultures of *Enterobacterales*, *Pseudomonas*, and *Acinetobacter* species. It was carried out as described by Pires et al. [26]. The reference strain *E. coli* ATCC^®^ 25922 was used as a negative control.

#### 2.3.2. Modified Carbapenem Inactivation Method (mCIM)

This test was used for the detection of carbapenemases in *Enterobacterales* and *P. aeruginosa*, as recommended by the CLSI guidelines 2020 [25]. A meropenem disk was submerged in a 2 mL TSB suspension of the tested isolate and incubated at 37 °C for 4 h. The disk was then transferred to the center of a Mueller–Hinton agar plate previously inoculated with *E. coli* ATCC^®^ 25922. Following overnight incubation, the inhibition zone around the disk was measured. Isolates showing a 6–15 mm zone diameter or the presence of pinpoint colonies within a 16–18 mm zone were considered CPs [25].

#### 2.3.3. EDTA-Modified Carbapenem Inactivation Method (eCIM)

The eCIM test was used together with mCIM to detect the production of MBLs in *Enterobacterales.* eCIM can only be performed for *Enterobacterales* isolates that give a positive mCIM test result. This test was performed and interpreted as described in CLSI guidelines 2020 [25].

### 2.4. Phenotypic Analysis Using Heatmap Signature

The antimicrobial resistance profiles and carbapenemase production results were used to generate a dendrogram showing heatmap signatures of the isolates to determine their phenotypic relatedness. It was generated by Morpheus online software (https://software.broadinstitute.org/morpheus/, accessed on 15 April 2021) using Euclidean distances.

### 2.5. Molecular Detection of Plasmid-Borne Carbapenemase Genes

#### 2.5.1. Extraction of Plasmid DNA from Carbapenem-Resistant Isolates

Overnight cultures of the CR-GNB isolates were grown in Luria Bertani broth containing 8 μg/mL meropenem as a selective pressure to enhance plasmid recovery. The extraction of plasmid DNA was carried out using GeneJet Plasmid Miniprep Kit Catalog number: K0502 for extraction or large-sized plasmids (Thermo Fisher Scientific, Waltham, MA, USA; https://www.thermofisher.com/order/catalog/product/K0502#/K0502, accessed on 15 April 2021). The extracted plasmids were analyzed via agarose gel electrophoresis (Sub-Cell^®^ GT Agarose Gel Electrophoresis Systems, Bio-Rad Laboratories, Hercules, CA, USA) and visualized by UV transilluminator (Benchtop 3UV transilluminator, UVP, LLC, Upland, CA, USA) [27].

#### 2.5.2. Amplification of Some Plasmid-Encoded Carbapenemase Genes

The extracted plasmids were used as templates for PCR using the appropriate primers synthesized by invitrogen^®^ (Thermo Fisher Scientific, Waltham, MA, USA), and Dream Taq^™^ Green PCR Master Mix (Thermo Fisher Scientific, Waltham, MA, USA). The annealing temperatures (T_a_) and primers for *bla*_KPC_, *bla*_NDM_, *bla*_VIM_, *bla*_OXA-48_, and *bla*_IMP_ genes are shown in Table 1, and the PCR conditions are listed in Table 2. The amplified PCR products were analyzed using agarose gel electrophoresis, and the sizes of the DNA fragments were determined by comparison to a 100 bp DNA ladder (GeneRuler 100 bp DNA ladder, Thermo Fisher Scientific, Waltham, MA, USA).

#### 2.5.3. Sequencing of PCR Products

Some PCR products of the amplified genes were sent for sequencing at Macrogen Inc. (Seoul, Korea) using an Applied Biosystems 3730XL sequencer. The assembly of the obtained forward and reverse sequence files into the final consensus sequence was completed using BioEdit v7.2.5 software [37]. The open reading frames (ORFs) of the final contigs were detected using FramePlot 4.0beta (http://nocardia.nih.go.jp/fp4/, accessed on 23 April 2021) [38]. The sequencing data were analyzed using the basic local alignment search tool BLASTn (https://blast.ncbi.nlm.nih.gov/Blast.cgi, accessed on 23 April 2021). The *bla*_OXA-48_, *bla*_NDM_, *bla*_KPC_, and *bla*_VIM_ nucleotide sequences were annotated and submitted into the NCBI GenBank database under the accession codes MW562895, MZ092838, MZ092839, and MZ092840, respectively.

#### 2.5.4. Transformation

*E. coli* DH5α is a standard strain that does not harbor any resistance genes. Competent *E. coli* DH5α cells were prepared according to the modified Hanahan method [39]. The extracted plasmids were transformed into competent *E. coli* DH5α to compare the phenotypic properties of the transformants with those of the parent clinical isolates. Chemical transformation was carried out as described in Sambrook and Russell [27]. Transformants were cultured on LB/ampicillin and LB/meropenem agar plates at concentrations of 100 µg/mL and 8 µg/mL, respectively. Untransformed *E. coli* DH5α was used as a negative control. Plasmid DNA was extracted from transformants that showed growth on LB/ampicillin and/or LB/meropenem plates, and was used as templates for PCR to confirm the presence of carbapenemase genes on the transformed MGEs, as well as to detect the presence of other antimicrobial resistance genes including ESBL genes (*bla*_SHV_, *bla*_CTX-M_, and *bla*_TEM_), and the *aac(6′)-Ib* gene which confers resistance to aminoglycosides. The annealing temperatures (T_a_) and primers for *bla*_SHV_, *bla*_CTX-M_, *bla*_TEM_, and *aac(6′)-Ib* genes are shown in Table 1, and the PCR conditions are in Table 2. Phenotypic tests including antimicrobial susceptibility testing, Blue-Carba, and mCIM, were also carried out for the transformants as previously described in this study, to detect the acquired phenotypic properties of the transformants.

### 2.6. Enterobacterial Repetitive Intergenic Consensus-PCR (ERIC-PCR) for Isolates Containing Plasmids

ERIC-PCR is a sequence analysis tool used for epidemiological analysis of bacterial isolates to determine their genetic relatedness. DNA template preparation of the isolates was performed as described by Doyle et al. [28] and the templates were used for PCR. The primers (Table 1) and conditions used for ERIC-PCR (Table 2) were described by Codjoe et al. [36]. ERIC fingerprinting data were transformed into a binary code depending on the presence (denoted 1) or absence (denoted 0) of each band. A dendrogram was generated by the unweighted pair group method with arithmetic average (UPGMA) and Ward’s hierarchical clustering routine using Statistical Package for the Social Sciences software IBM^®^ SPSS^®^ version 23 (IBM Corp., Armonk, NY, USA) [40,41], and a proximity matrix by Jaccard measure was generated to calculate the similarity index of the isolates.

### 2.7. Statistical Analysis

Frequency tables, crosstabs, dendrogram construction, similarity index calculation, and chi-square analysis were carried out using Statistical Package for the Social Sciences software IBM^®^ SPSS^®^ version 23 (IBM Corp., Armonk, NY, USA) [41].

## 3. Results

### 3.1. Collection of Clinical Isolates

Based on the hospitals’ records, a total of 256 non-duplicate GNB clinical isolates were collected from patients with cUTIs (patients having colony count of uropathogen >10^5^ cfu/mL in the urine sample and one or more risk factors of cUTIs). Among the patient population, 111 (43.4%) were females, while 145 (56.6%) were males. Based on the hospitals’ records, the chronological age of the patients ranged from 3 months to 82 years, 40 of which (15.6%) were below the age of 18 years, 96 (37.5%) were in the age range of 18–40 years, 56 (21.9%) were in the age range of 41–60 years, and 64 (25%) were above the age of 60 years.

### 3.2. Identification of the Collected GNB Clinical Isolates

Out of the collected 256 GNB clinical isolates, 210 (82%) were identified as *Enterobacterales*, including 96 (37.5%) *E. coli*, 87 (34%) *Klebsiella* spp., 18 (7%) *Proteus* spp., and 9 (3.5%) *Enterobacter* spp. The remaining GNB isolates included 31 (12%) *Pseudomonas* spp., and 15 (6%) *Acinetobacter* spp.

### 3.3. Antimicrobial Susceptibility of the Collected Isolates

**Figure 1:**
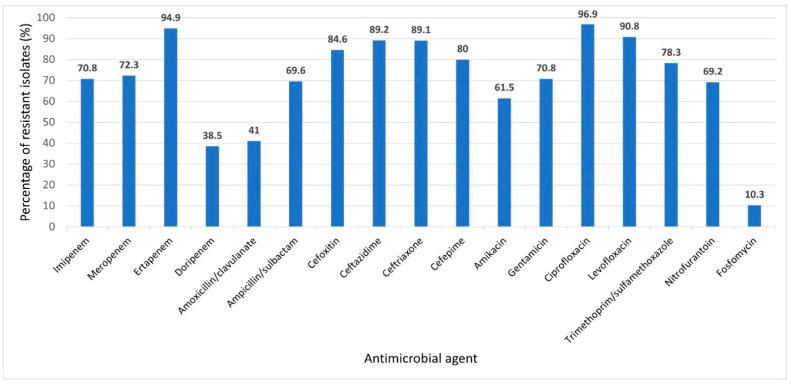
Prevalence of antimicrobial resistance of 65 carbapenem-resistant Gram-negative bacterial uropathogens to various tested antimicrobial agents. Prevalence was expressed as percent of resistant isolates relative to the total tested bacterial species for each antimicrobial agent.

Among the 256 GNB isolates, 65 (25.4%) showed resistance to one or more of the tested carbapenems; they were categorized as carbapenem-resistant Gram-negative bacterial (CR-GNB) isolates and were selected for further studying. The CR-GNB isolates were *Klebsiella* spp. (28; 43.1%), including 22 *K. pneumoniae* and 6 *K. terrigena*, *P*. *aeruginosa* (19; 29.2%), *Acinetobacter baumannii* (8; 12.3%), *E*. *coli* (6; 9.2%), *Proteus mirabilis* (2; 3.1%), and *Enterobacter cloacae* (2; 3.1%). The results of antimicrobial susceptibility testing showed that 64 out of 65 CR-GNB isolates were MDR, showing the highest resistance rates to ciprofloxacin (96.9%), ertapenem (94.7%), levofloxacin (90.8%), ceftazidime (89.2%), and ceftriaxone (89.1%). The lowest rates of resistance were to fosfomycin (10.5%), followed by doripenem (38.5%). The percentage of antimicrobial resistance of the 65 CR-GNB isolates are demonstrated in Figure 1, and their resistance patterns relative to identity are shown in Table 3. The results of antibiogram analysis of the CR-GNB isolates in this study, as well as the identity of the isolates, are included in the supplementary material (Supplementary Tables S1–S3).

### 3.4. Phenotypic Detection of CPs

**Figure 2:**
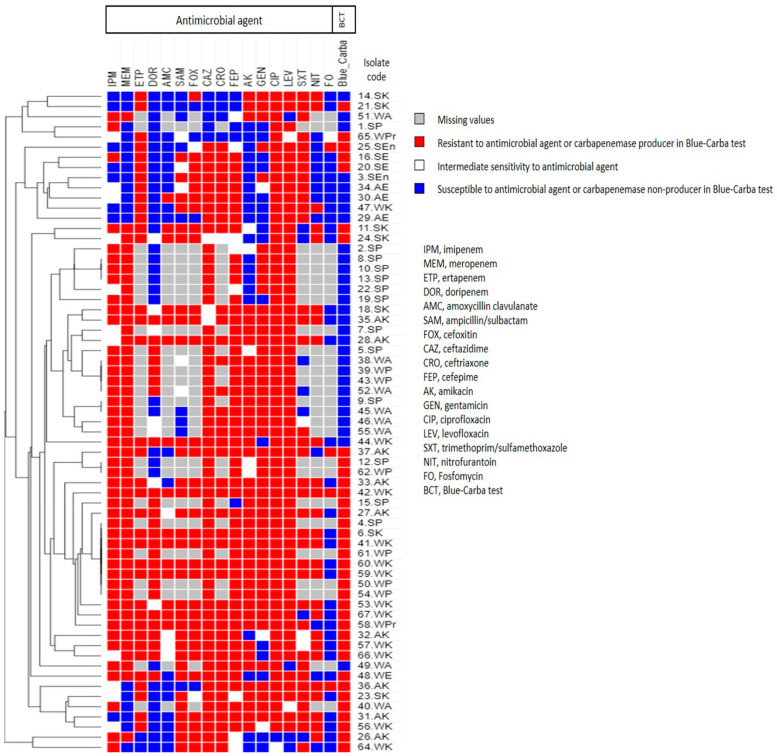
Heatmap of carbapenem-resistant Gram-negative bacterial isolates (*n* = 65) in this study based on their antimicrobial resistance patterns and carbapenemase enzyme production (Blue-Carba test results). This heatmap was generated by using Morpheus online software using Euclidean distances (https://software.broadinstitute.org/morpheus/, accessed on 15 April 2021).

Carbapenemase enzyme production by the CR-GNB isolates (*n* = 65) was determined phenotypically using the Blue-Carba and mCIM tests. The production of MBLs was detected using the eCIM test, the results of which are shown in Table 4. Based on the results of Blue-Carba test, 36 out of 65 (55.4%) CR-GNB isolates were CPs, 29 were carbapenemase-producing *Enterobacteriaceae* (CPE) as confirmed by mCIM test. The results of antimicrobial resistance and carbapenemase production were used to generate a dendrogram showing heatmap signature of the isolates (Figure 2). This was in hopes of providing more insight on the phenotypic relatedness of the isolates. The heatmap signature of five pairs of *P. aeruginosa* isolates and one pair of *A. baumannii* isolates were found to be similar based on their phenotypic properties.

### 3.5. Extraction of Plasmid DNA and Amplification of Carbapenemase Genes

Out of the 65 CR-GNB isolates, 31 (47.7%) harbored plasmids. Agarose gel electrophoresis of some of the extracted plasmids is shown in Supplementary Figure S1. The plasmid extracts were used as templates for PCR amplification of the carbapenemase genes *bla*_KPC_, *bla*_NDM_, *bla*_VIM_, *bla*_OXA-48_, and *bla*_IMP_. The PCR results of multiplex *bla*_VIM_/*bla*_OXA-48_ revealed that *bla*_OXA-48_ gene was detected in the plasmids of 24 (77.4%) of the tested isolates, while *bla*_VIM_ gene was detected in 8 (25.8%) of the isolates. The PCR results of multiplex *bla*_KPC_/*bla*_NDM_ showed that both *bla*_KPC_ and *bla*_NDM_ genes were co-present in one (3.2%) isolate. The *bla*_IMP_ gene was not detected in any of the plasmid extracts. The results of agarose gel electrophoresis of multiplexes *bla*_KPC_/*bla*_NDM_ and *bla*_VIM_/*bla*_OXA-48_ of some tested CR-GNB isolates are demonstrated in Supplementary Figures S2 and S3, respectively.

### 3.6. Transformation of Plasmids into Competent E. coli DH5α

Plasmid extracts that harbored any of the tested carbapenemase genes were transformed into competent *E. coli* DH5α to compare the phenotypic properties of the transformants to those of the parent clinical isolates. Transformants were cultured on LB/ampicillin and LB/meropenem agar plates at concentrations of 100 µg/mL and 8 µg/mL, respectively. Untransformed *E. coli* DH5α was used as a negative control.

The successfully transformed MGEs in the current study were all extracted from *K. pneumoniae* clinical isolates, transformation from other clinical isolates was not successful. One transformant (code: TS24.SK) showed growth on both LB/ampicillin and LB/meropenem plates, indicating successful transformation of a MGE carrying at least one carbapenem resistance gene. A single colony of this transformant was picked and subcultured on an LB/meropenem plate. Visible growth was observed after overnight incubation. Antimicrobial susceptibility testing revealed the newly acquired properties of the transformant, where it acquired resistance to meropenem, ertapenem, ampicillin/sulbactam, cefoxitin, ceftazidime, ciprofloxacin, and levofloxacin, and intermediate sensitivity to imipenem, ceftriaxone, and cefepime.

Three other transformants (codes: TS37.AK, TS59.WK, and TS66.WK) showed growth on LB/ampicillin agar plates, indicating successful transformation of an MGE carrying at least one antimicrobial resistance gene. A single colony of each transformant was picked and subcultured on LB/ampicillin plates to increase the plasmid copy number by selective pressure, then further subcultured on LB/meropenem plates. Weak but visible growth was observed on the LB/meropenem plates after overnight incubation. Antimicrobial susceptibility testing revealed that these transformants acquired resistance to ampicillin/sulbactam, cefoxitin, and ceftazidime, and intermediate sensitivity to ceftriaxone. Both TS37.AK and TS59.WK acquired intermediate sensitivity to imipenem, while TS66.WK became resistant to it.

**Figure 3:**
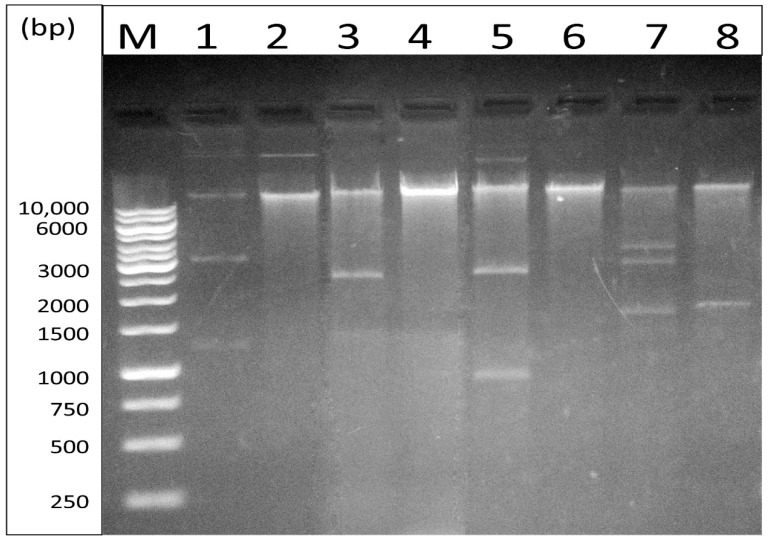
Agarose gel electrophoresis of plasmid DNA extracted from some carbapenem-resistant Gram-negative bacterial isolates and their corresponding transformants; lane M, a Gene Ruler 1 kb ladder; lane 1, clinical isolate 37.AK; lane 2, transformant TS37.AK; lane 3, clinical isolate 59.WK; lane 4, transformant TS59.WK; lane 5, clinical isolate 66.WK; lane 6, transformant TS66.WK; lane 7, clinical isolate 24.SK; lane 8, transformant TS24.SK.

Plasmid DNA was extracted from the transformants (*n* = 4). Agarose gel electrophoresis of plasmid DNA extracted from CR-GNB isolates and their corresponding transformants are shown in Figure 3. Plasmid DNA from the transformants was used as templates for PCR to confirm the presence of carbapenemase genes in the transformants, as well as to detect the presence of other antimicrobial resistance genes including ESBL genes (*bla*_SHV_, *bla*_CTX-M_, and *bla*_TEM_), and the *aac(6′)-Ib* gene which confers resistance to aminoglycosides. Untransformed *E. coli* DH5α was used as a negative control.

The results of PCR and gel electrophoresis confirmed that all the transformants were carrying *bla*_OXA-48_ gene, two of which carried the *bla*_CTX-M_ gene, one carried the *bla*_TEM_ gene, and one carried the *aac(6′)-Ib gene.* All transformants also gave positive results with Blue-Carba and mCIM tests. Phenotypic properties of the transformants were compared to those of untransformed *E. coli* DH5α, and the parent clinical isolates (24.SK, 37.AK, 59.WK, 66.WK, which were identified as *K. pneumoniae*) from which the plasmids were extracted and transformed, as shown in Table 5. Phenotypic and genotypic characteristics of the transformed plasmids are summarized in Table 6.

### 3.7. Genotyping of CR-GNB Containing Plasmids

ERIC-PCR was performed for the 31 CR-GNB isolates harboring plasmids to determine their genetic relatedness. The tested isolates included 23 *Klebsiella* spp., 3 *E. coli*, 3 *P. aeruginosa*, and 2 *A. baumannii*. The ERIC-PCR gel analysis exhibited a range from 3 to 13 bands between the sizes of 111 to 1778 bp (Supplementary Figure S4).

**Figure 4:**
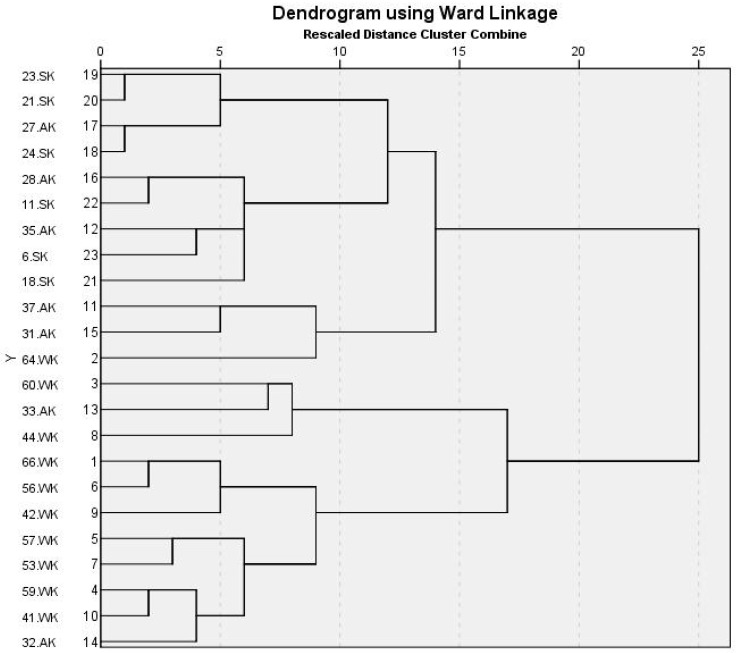
Dendrogram generated from ERIC-PCR genomic DNA products of 23 carbapenem-resistant *Klebsiella* spp. isolates harboring plasmids.

As shown in Figure 4, the dendrogram generated from ERIC-PCR genomic DNA products of *Klebsiella* spp. isolates reveals that the tested isolates were not clonal, which ensures the genetic diversity of the isolates. However, based on the calculated Jaccard similarity index, isolates 21.SK and 23.SK were found to be 100% similar, as well as isolates 24.SK and 27.AK, although they were all collected from different patients. Aside from these four isolates, all the other plasmid-bearing isolates in this study were genetically dissimilar, as confirmed by the calculated similarity index of the isolates.

### 3.8. Statistical Analysis

Categorical variables were analyzed using chi-square analysis to detect statistically significant associations between phenotypic and genotypic properties of the tested isolates. Significance was two-sided, and a value of *p* < 0.05 was considered statistically significant.

The results have revealed significant associations between the phenotypic resistance of the isolates to amoxicillin/clavulanic acid and the presence of *bla*_VIM_ and *bla*_OXA_ genes on their plasmids, with Pearson chi-square values of 0.048 and 0.01, respectively. There was also a significant association between the phenotypic resistance of the isolates to ampicillin/sulbactam and the presence of the *bla*_VIM_ gene on their plasmids, with a Pearson chi-square value of 0.004.

## 4. Discussion

GNB are common causes of nosocomial and community-acquired UTls [42]. Owing to limited therapeutic options, UTls caused by antibiotic-resistant GNB are an increasing concern [43]. Such pathogens are prone to acquiring genes encoding multiple mechanisms of antibiotic resistance, including ESBLs and carbapenemases [44]. CR occurs mainly among GNB, such as *Enterobacterales*, *P. aeruginosa*, and *A. baumannii*, and may either be intrinsic or conferred by transferable carbapenemase-encoding genes. Thus, our aim was to detect the carbapenemase-producing ability of GNB isolated from urine specimens of cUTI patients admitted to two of the most important tertiary care hospitals in Cairo, Egypt, to determine the prevalence of carbapenemase genes among plasmid-bearing isolates, and to explore the possible transferability of such resistance to other bacterial host(s).

Out of 210 *Enterobacterales* in the current study, 38 (18.1%) were carbapenem-resistant *Enterobacteriaceae* (CRE). Contrary to our results, a study on the epidemiology of CRE in Egyptian intensive care units reported that 117/236 (49.6%) *Enterobacterales* isolates from urine specimens were CRE, suggesting the presence of healthcare transmission [45]. Antimicrobial susceptibility testing showed that 64 out of 65 CR-GNB isolates were MDR, which was in accordance to previously published studies that underscore the considerable resistance of many CPs [46,47,48].

Among the collected uropathogens in this study, 29 out of 210 (13.8%) *Enterobacterales* were CPE. A study by Woodford et al. reported that the rate of confirmed CPE among urinary GNB isolates from five UK laboratories participating in the study was 0.13% [49]. The proportion of CPE was 2.3% in another study by Giani et al. on urinary GNB isolates from five Italian hospitals [50]. At a referral hospital in northwest Ethiopia, 5/183 (2.73%) *Enterobacterales* isolates from UTI patients were CPE [51]. These rates are substantially lower than the one observed in the current study (13.8%), indicating weak infection control strategies. This might be attributed to the overuse of broad-spectrum antimicrobials as empiric treatments for all gravely ill patients, out of fear of missing an individual with a highly resistant pathogen.

In the present study, PCR amplification was used to detect some plasmid-borne carbapenemase genes including *bla*_KPC_, *bla*_NDM_, *bla*_VIM_, *bla*_OXA-48_, and *bla*_IMP._ The most prevalent of which was *bla*_OXA-48_ gene (77.4%), followed by *bla*_VIM_ gene (25.8%). Both *bla*_KPC_ and *bla*_NDM_ genes were co-present in one isolate. The *bla*_IMP_ gene was not carried in the plasmids of any of the tested isolates, which agreed with the results of other studies from Egypt reporting the absence of *bla*_IMP_ gene from the tested CR-GNB isolates [48,52]. However, this does not negate the presence of IMP carbapenemase in Egypt, as it was reported to be harbored by *P. aeruginosa* isolates in Egypt in 2017 [53].

Plasmid DNA extracted from CR-GNB isolates was transformed into competent *E. coli* DH5α. Four transformants were able to grow on LB/ampicillin agar plates two successive generations, then on LB/meropenem plates, indicating successful transformation of one or more MGEs carrying resistance genes. The transformed MGEs were extracted using the GeneJet Plasmid Miniprep Kit, and were found to carry the *bla*_OXA-48_ gene by PCR amplification, which in turn confirmed the possibility of horizontal transfer of carbapenemase-encoding genes. In accordance with our findings, a study performed by Göttig et al. established the in vivo transfer of a plasmid carrying a *bla*_OXA-48_ gene from *K. pneumoniae* to *E. coli* in the gut of an infected patient [54].

Antibiogram analysis was carried out for the transformants. The results of this revealed the newly acquired properties of the transformants, where one transformant acquired resistance to meropenem, ertapenem, ampicillin/sulbactam, cefoxitin, ceftazidime, ciprofloxacin, and levofloxacin, and intermediate sensitivity to imipenem, ceftriaxone, and cefepime. The remaining three transformants showed resistance to ampicillin/sulbactam, cefoxitin, and ceftazidime, and intermediate sensitivity to ceftriaxone, two of which acquired intermediate sensitivity to imipenem, while one acquired imipenem resistance. It is well known that the OXA-48 enzyme has a high capacity for the hydrolysis of penicillins, a low capacity for the hydrolysis of carbapenems, and does not hydrolyze broad-spectrum cephalosporins [21]; thus, the acquired resistance of the transformants to cephalosporins and carbapenems may be attributed to other resistance determinants carried on the same OXA-encoding plasmid, that was transferred to the previously susceptible *E. coli* DH5α.

All transformants gave a positive result with Blue-Carba and mCIM tests, indicating the production of carbapenemase enzymes. It is worth noting that three of the transformants initially failed to grow on LB/meropenem plates, and only grew on LB/ampicillin. The copy number of the plasmids might have increased on LB/ampicillin plates, allowing the sufficient replication of the CR genes, and enabling the transformants to grow on LB/meropenem plates upon subculturing from LB/ampicillin plates.

Our future perspectives include whole plasmid sequencing of the successfully transformed plasmids to detect all the other resistance determinants carried on such plasmids, in addition to fully characterizing these transferable plasmids.

## 5. Conclusions

This study shows that resistance to carbapenems, one of the last resort classes of antibiotics, became prevalent and distributed among Gram-negative bacterial uropathogens in two major tertiary care hospitals in Cairo, Egypt. This type of resistance can be transferred horizontally to other bacterial hosts causing limitations and challenges in treatment options of bacterial infections. Antibiotic stewardship programs must be implemented to reduce the emergence and spread of CR and improve the outcomes of infectious diseases treatment programs.

## Figures and Tables

**Table 1:** Primers used in this study, expected PCR product sizes, and annealing temperatures (Ta).

PCR Reaction	Gene	Primer	Primer Sequence (5′ → 3′)	Expected PCR Product Size (bp)	T_a_ (°C)	References
Multiplex	*bla*_KPC_	P_f_	TGTCACTGTATCGCCGTC	1011	50	[28]
		P_r_	CTCAGTGCTCTACAGAAAACC			
	*bla*_NDM_	P_f_	GGTTTGGCGATCTGGTTTTC	621		[29,30]
		P_r_	CGGAATGGCTCATCACGAT			
Multiplex	*bla*_VIM_	P_f_	TCTACATGACCGCGTCTGTC	748	50	[31]
		P_r_	TGTGCTTTGACAACGTTCGC			
	*bla*_OXA-48_	P_f_	GCGTGGTTAAGGATGAACAC	438		[28,30]
		P_r_	CATCAAGTTCAACCCAACCG			
Monoplex	*bla*_IMP_	P_f_	CTACCGCAGCAGAGTCTTTG	587	50	[32]
		P_r_	AACCAGTTTTGCCTTACCAT			
Multiplex	*aac(6′)-Ib*	P_f_	TTGCGATGCTCTATGAGTGG	358	49	[33]
		P_r_	CGTTTGGATCTTGGTGACCT			
	*bla*_SHV_	P_f_	GGTTATGCGTTATATTCGCC	867		[34]
		P_r_	TTAGCGTTGCCAGTGCTC			
Multiplex	*bla*_CTX-M_	P_f_	CGCTTTGCGATGTGCAG	550	51	[35]
		P_r_	ACCGCGATATCGTTGGT			
	*bla*_TEM_	P_f_	ATGAGTATTCAACATTTCCG	867		[34]
		P_r_	CTGACAGTTACCAATGCTTA			
ERIC-PCR		P_f_	AAGTAAGTGACTGGGGTGAGCG	Variable	45	[36]
		P_r_	ATGTAAGCTCCTGGGGATTCAC			

Notes: *bla*_KPC_, *bla*_NDM_, *bla*_VIM_, *bla*_OXA-48_, and *bla*_IMP_ genes code for KPC, NDM, VIM, OXA-48-like, and IMP carbapenemases, respectively, *aac(6ʹ)-Ib* gene codes for aminoglycoside 6ʹ-N-acetyltransferase type Ib, *bla*_CTX-M_, *bla*_SHV_, and *bla*_TEM_ genes code for ESBLs. Abbreviations: P_f_, forward primer; P_r_, reverse primer; T_a_, annealing temperature.

**Table 2:** Conditions of PCR cycles (thermocycler input data).

PCR Reaction	Primary Denaturation	Secondary Denaturation	Annealing	Extension	No. of Cycles	Final Extension
Multiplex *bla*_KPC_/*bla*_NDM_	95 °C 4 min	95 °C 30 s	50 °C 45 s	72 °C 1 min	30	72 °C 10 min
Multiplex *bla*_VIM_/*bla*_OXA-48_	95 °C 4 min	95 °C 30 s	50 °C 45 s	72 °C 1 min	30	72 °C 10 min
Monoplex *bla*_IMP_	95 °C 4 min	95 °C 30 s	50 °C 45 s	72 °C 1 min	30	72 °C 10 min
Multiplex *aac(6′)-Ib/bla*_SHV_	95 °C 4 min	95 °C 30 s	49 °C 45 s	72 °C 1 min	30	72 °C 10 min
Multiplex *bla*_CTX-M_/*bla*_TEM_	95 °C 4 min	95 °C 30 s	51 °C 45 s	72 °C 1 min	30	72 °C 10 min
ERIC-PCR	95 °C 15 min	94 °C 30 s	45 °C 45 s	72 °C 7 min	45	72 °C 10 min

Notes: *bla*_KPC_, *bla*_NDM_, *bla*_VIM_, *bla*_OXA-48_, and *bla*_IMP_ genes code for KPC, NDM, VIM, OXA-48-like, and IMP carbapenemases, respectively, *aac(6ʹ)-Ib* gene codes for aminoglycoside 6ʹ-N-acetyltransferase type Ib, *bla*_CTX-M_, *bla*_SHV_, and *bla*_TEM_ genes code for ESBLs.

**Table 3:** Resistance patterns to various antimicrobial agents among different tested carbapenem-resistant Gram-negative bacterial isolates (*n* = 65).

Antimicrobial Class	Antimicrobial Agent	Percentage of Resistance (%)					
		*Klebsiella* spp. (*n* = 28)	*P. aeruginosa* (*n* = 19)	*A. baumannii* (*n* = 8)	*E. coli* (*n* = 6)	*Proteus mirabilis* (*n* = 2)	*Enterobacter cloacae* (*n* = 2)
Carbapenems	Imipenem (10 µg)	64.3	89.5	100	33.3	50	0
	Meropenem (10 µg)	71.4	94.7	87.5	16.7	50	0
	Ertapenem (10 µg)	92.9	ND	ND	100	100	100
	Doripenem (10 µg)	46.4	42.1	25	16.7	50	0
β-lactam combination agents	Amoxicillin/clavulanic acid (30 µg)	46.4	ND	ND	16.7	50	0
	Ampicillin/sulbactam (20 µg)	89.3	ND	25	50	50	50
Cephalosporins	Cefoxitin (30 µg)	89.3	ND	ND	83.3	50	50
	Ceftazidime (30 µg)	85.7	94.7	87.5	100	50	100
	Ceftriaxone (30 µg)	89.3	ND	87.5	100	50	100
	Cefepime (30 µg)	78.6	78.9	87.5	100	50	50
Aminoglycosides	Amikacin (30 µg)	78.6	47.4	100	0	50	0
	Gentamicin (10 µg)	64.3	89.5	100	0	50	100
Fluoroquinolones	Ciprofloxacin (5 µg)	92.9	100	100	100	100	100
	Levofloxacin (5 µg)	92.9	100	62.5	100	50	100
Folate pathway inhibitors	Trimethoprim/sulfamethoxazole (25 µg)	78.6	ND	50	100	100	100
Nitrofurans	Nitrofurantoin (300 µg)	92.9	ND	ND	0	0	0
Fosfomycins	Fosfomycin (200 µg)	10.7	ND	ND	0	0	50

Abbreviations: ND, not determined.

**Table 4:** Phenotypic detection of carbapenemase production in carbapenem-resistant Gram-negative bacterial isolates.

Tested Isolates	Blue-Carba Test (*n* = 65) [26]		Modified Carbapenem Inactivation Method (mCIM) (*n* = 57) [25]		EDTA-Modified Carbapenem Inactivation Method (eCIM) (*n* = 29) [25]	
	No. of CPs/Total No. of Tested Isolates	%	No. of CPs/Total No. of Tested Isolates	%	No. of MBL Producers/Total No. of Tested Isolates	%
*Klebsiella* spp.	22/28	78.6	23/28	82.1	12/23	52.2
*P. aeruginosa*	7/19	36.8	2/19	10.5	ND	ND
*A. baumannii*	1/8	12.5	ND	ND	ND	ND
*E. coli*	3/6	50	4/6	66.7	3/4	75
*Proteus mirabilis*	2/2	100	1/2	50	0/1	0
*Enterobacter cloacae*	1/2	50	1/2	50	1/1	100

Notes: Blue-Carba test is a phenotypic test for the detection of carbapenemase production in *Enterobacterales*, *Pseudomonas* spp., and *Acinetobacter* spp. Modified carbapenem inactivation method (mCIM) test is a phenotypic test for the detection of carbapenemase production in *Enterobacterales* and *Pseudomonas* spp., but not *Acinetobacter* spp. EDTA-modified carbapenem inactivation method (eCIM) is carried out only for *Enterobacterales* that give a positive result in mCIM. CPs, carbapenemase-producers; MBL, metallo-β-lactamase; ND, not determined.

**Table 5:** Phenotypic properties of transformants, their corresponding *K. pneumoniae* parent clinical isolates, and untransformed *E. coli* DH5α.

	TS24.SK	24.SK	TS37.AK	37.AK	TS59.WK	59.WK	TS66.WK	66.WK	*E. coli* DH5α
Antimicrobial susceptibility testing									
Imipenem (10 µg)	I	I	I	R	I	R	I	I	S
Meropenem (10 µg)	R	R	S	R	S	R	S	R	S
Ertapenem (10 µg)	R	R	S	R	S	R	S	R	S
Doripenem (10 µg)	S	I	S	S	S	R	S	R	S
Amoxicillin/clavulanic acid (30 µg)	S	R	S	S	S	R	S	I	S
Ampicillin/sulbactam (20 µg)	R	R	R	R	R	R	R	R	S
Cefoxitin (30 µg)	R	R	R	R	R	R	R	R	S
Ceftazidime (30 µg)	R	R	R	R	R	R	R	R	S
Ceftriaxone (30 µg)	I	I	I	R	I	R	R	R	S
Cefepime (30 µg)	SDD	SDD	S	R	S	R	SDD	R	S
Amikacin (30 µg)	S	S	S	R	S	R	S	R	S
Gentamicin (10 µg)	S	S	S	R	S	R	S	S	S
Ciprofloxacin (5 µg)	R	R	S	R	S	R	S	R	S
Levofloxacin (5 µg)	R	R	S	R	S	R	S	R	S
Trimethoprim/sulfamethoxazole (25 µg)	S	S	S	R	S	R	S	R	S
Nitrofurantoin (300 µg)	S	R	S	S	S	R	S	R	S
Fosfomycin (200 µg)	S	S	S	R	S	S	S	S	S
**Blue-Carba test**									
	+	+	+	+	+	+	+	+	-
**Modified carbapenem inactivation method (mCIM)**									
	+	+	+	+	+	+	+	+	-

Notes: TS24.SK, TS37.AK, TS59.WK, and TS66.WK are transformants of *K. pneumoniae* clinical isolates 24.SK, 37.AK, 59.WK, and 66.WK, respectively. Untransformed *E. coli* DH5α was used as negative control. Abbreviations: S, sensitive; R; resistant; I, intermediate sensitivity; SDD, susceptible dose-dependent (a breakpoint category for which the susceptibility of an isolate depends on the dosing regimen used).

**Table 6:** Phenotypic and genotypic characteristics of the transformed plasmids.

Parental Strain	Transformant Code	Plasmid Code	Acquired Transformant Phenotype		Plasmid Genotype	Plasmid Size in Gel
24.SK	TS24.SK	pKPT24	Imipenem (10 µg)	I	*bla*_OXA-48_/*bla*_CTX-M_	2 plasmid bands: Large plasmid: >10 kb Small plasmid: ≈1700 bp
			Meropenem (10 µg)	R		
			Ertapenem (10 µg)	R		
			Ampicillin/sulbactam (20 µg)	R		
			Cefoxitin (30 µg)	R		
			Ceftazidime (30 µg)	R		
			Ceftriaxone (30 µg)	I		
			Cefepime (30 µg)	SDD		
			Ciprofloxacin (5 µg)	R		
			Levofloxacin (5 µg)	R		
37.AK	TS37.AK	pKPT37	Imipenem (10 µg)	I	*bla*_OXA-48_	2 plasmid bands: Large plasmid: >10 kb Small plasmid: >10 kb
			Ampicillin/sulbactam (20 µg)	R		
			Cefoxitin (30 µg)	R		
			Ceftazidime (30 µg)	R		
			Ceftriaxone (30 µg)	I		
59.WK	TS59.WK	pKPT59	Imipenem (10 µg)	I	*bla*_OXA-48_/*bla*_TEM_/*bla*_aac(6′)-Ib_	1 plasmid band: >10 kb
			Ampicillin/sulbactam (20 µg)	R		
			Cefoxitin (30 µg)	R		
			Ceftazidime (30 µg)	R		
			Ceftriaxone (30 µg)	I		
66.WK	TS66.WK	pKPT66	Imipenem (10 µg)	I	*bla*_OXA-48_/*bla*_CTX-M_	1 plasmid band: >10 kb
			Ampicillin/sulbactam (20 µg)	R		
			Cefoxitin (30 µg)	R		
			Ceftazidime (30 µg)	R		
			Ceftriaxone (30 µg)	R		
			Cefepime (30 µg)	SDD		

Notes: *bla*_OXA-48_ gene codes for OXA-48-like carbapenemase; *bla*_CTX-M_ and *bla*_TEM_ genes code for ESBLs; *aac(6ʹ)-Ib* gene codes for aminoglycoside 6ʹ-N-acetyltransferase type Ib.

## Data Availability

All the data supporting the findings are included in the manuscript and supplementary material.

## Supplementary Materials

The following are available online at https://www.mdpi.com/article/10.3390/biology10090889/s1, Figure S1: Agarose gel electrophoresis of plasmid DNA extracted from some carbapenem-resistant Gram-negative bacterial isolates; lane M, a Gene Ruler 1 kb ladder; lanes 1 through 8 are positive for plasmid bands; Figure S2: Agarose gel electrophoresis of multiplex-PCR amplification of *bla*_KPC_/*bla*_NDM_ genes in some carbapenem-resistant Gram-negative isolates, lane M, a Gene Ruler 100 bp ladder; lane 1 is positive for *bla*_KPC_ and *bla*_NDM_ with expected sizes of 1011 and 621 bp, respectively; lanes, 2,3, and 4 were negative; lane 5 is negative control. Arrows indicate positive bands; Figure S3: Agarose gel electrophoresis of multiplex-PCR amplification of *bla*_VIM_/*bla*_OXA-48_ genes in some carbapenem-resistant Gram-negative isolates, lane M, a Gene Ruler 100 bp ladder; lanes 2, 5, 7 are positive for *bla*_VIM_ with expected size of 748 bp; lanes 1, 2, 3, 4, 5, 6, 7, 8, 9 are positive for *bla*_OXA-48_ with expected size of 438 bp; lane 10 is negative control; Figure S4. Agarose gel electrophoresis of ERIC fingerprints of 31 carbapenem-resistant Gram-negative bacterial isolates harboring plasmids on agarose gel electrophoresis; lane L, DNA size marker; lanes 1 through 31, numbered fingerprints. By visual inspection, samples 8 and 9 show close relatedness on the gel; Table S1: Antibiogram analysis of carbapenem-resistant *Enterobacteriaceae* isolates (*n* = 38); Table S2: Antibiogram analysis of carbapenem-resistant *Pseudomonas aeruginosa* isolates (*n* = 19); Table S3: Antibiogram analysis of carbapenem-resistant *Acinetobacter baumannii* isolates (*n* = 8)).
