# Supplementary material for: OXA-48 Carbapenemase-Encoding Transferable Plasmids of Klebsiella pneumoniae Recovered from Egyptian Patients Suffering from Complicated Urinary Tract Infections
Source: Biology (Basel). 2021 Sep 9;10(9):889. doi: 10.3390/biology10090889 (PMC8469419; doi:10.3390/biology10090889)
Supplement: Supplementary file 1 [file biology-10-00889-s001.zip › biology-1307152-supplementary.pdf]

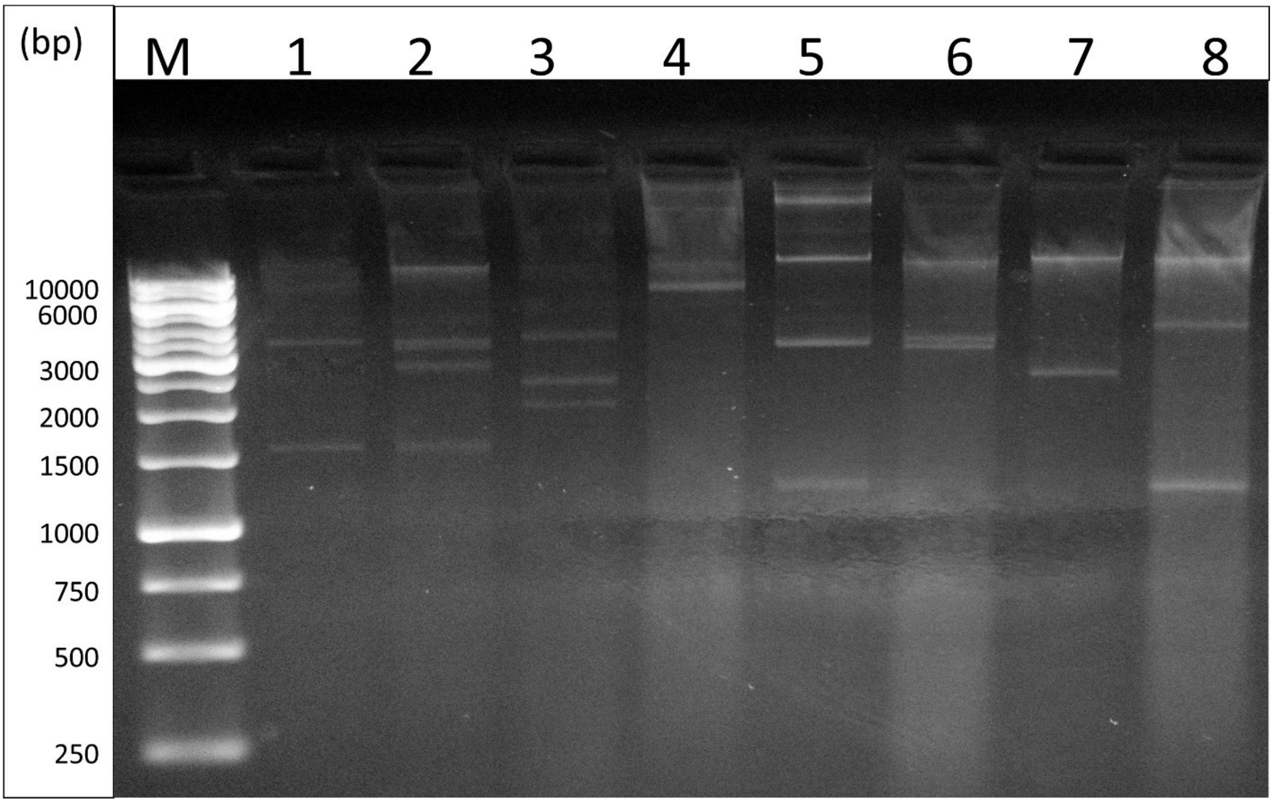

Figure S1. Agarose gel electrophoresis of plasmid DNA extracted from some carbapenem-resistant Gram-negative bacterial isolates; lane M, a gene Ruler 1 kb ladder; lanes 1 through 8 are positive for plasmid bands.

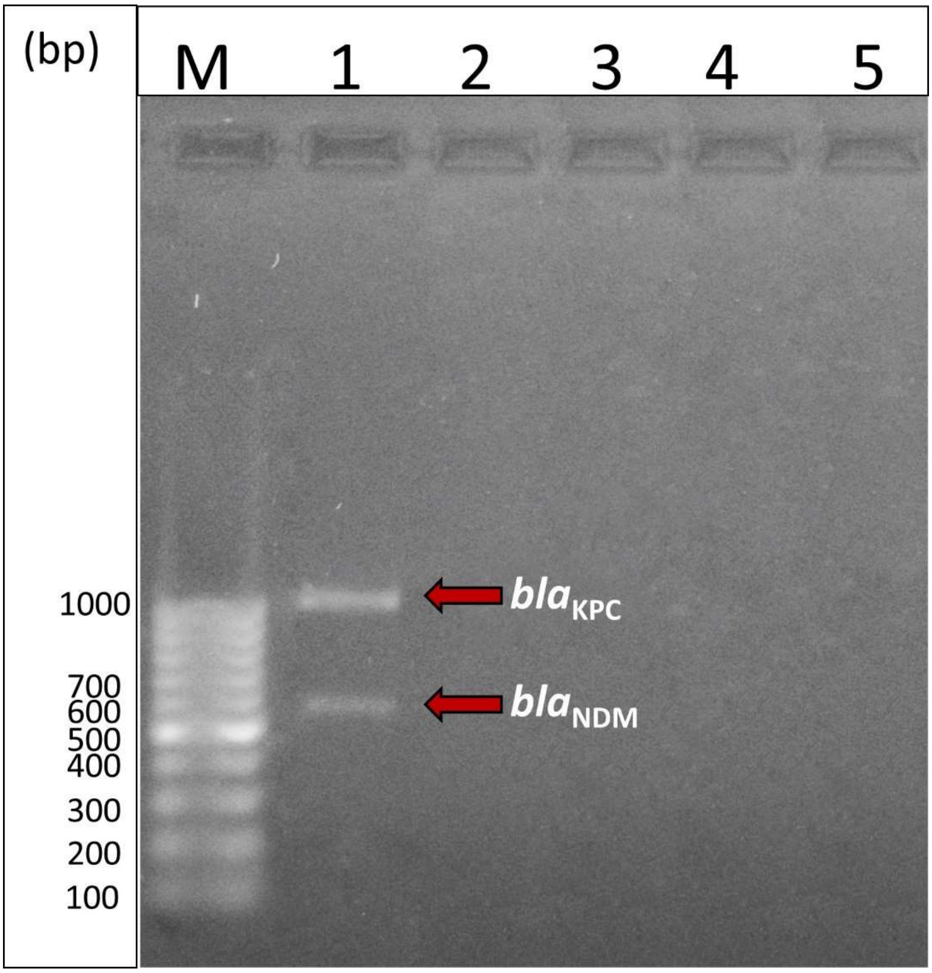

Figure S2. Agarose gel electrophoresis of multiplex-PCR amplification of *bla*$_{KPC}$ /*bla*$_{NDM}$ genes in some carbapenem-resistant Gram-negative isolates**,** lane M, a gene Ruler 100 bp ladder; lane 1 is positive for *bla*$_{KPC}$ and *bla*$_{NDM}$ with expected sizes of 1011 and 621 bp, respectively; lanes, 2,3, and 4 were negative; lane 5 is negative control. Arrows indicate positive bands.

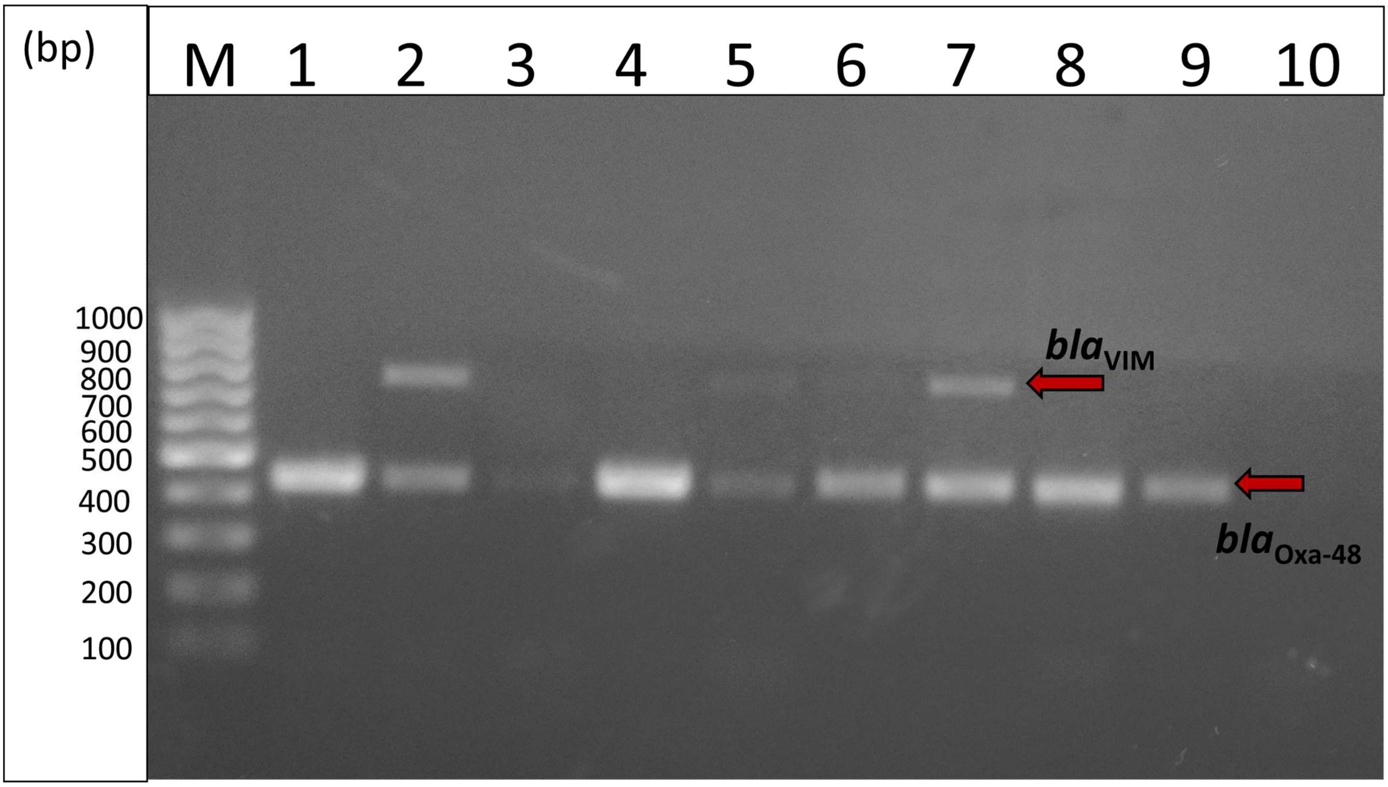

Figure S3. Agarose gel electrophoresis of multiplex-PCR amplification of bla_{VIM} /bla_{OXA-48} genes in some carbapenem-resistant Gram-negative isolates**,** lane M, a gene Ruler 100 bp ladder; lanes 2, 5, 7 are positive for bla_{VIM} with expected size of 748 bp; lanes 1, 2, 3, 4, 5, 6, 7, 8, 9 are positive for bla_{OXA-48} with expected size of 438 bp; lane 10 is negative control.

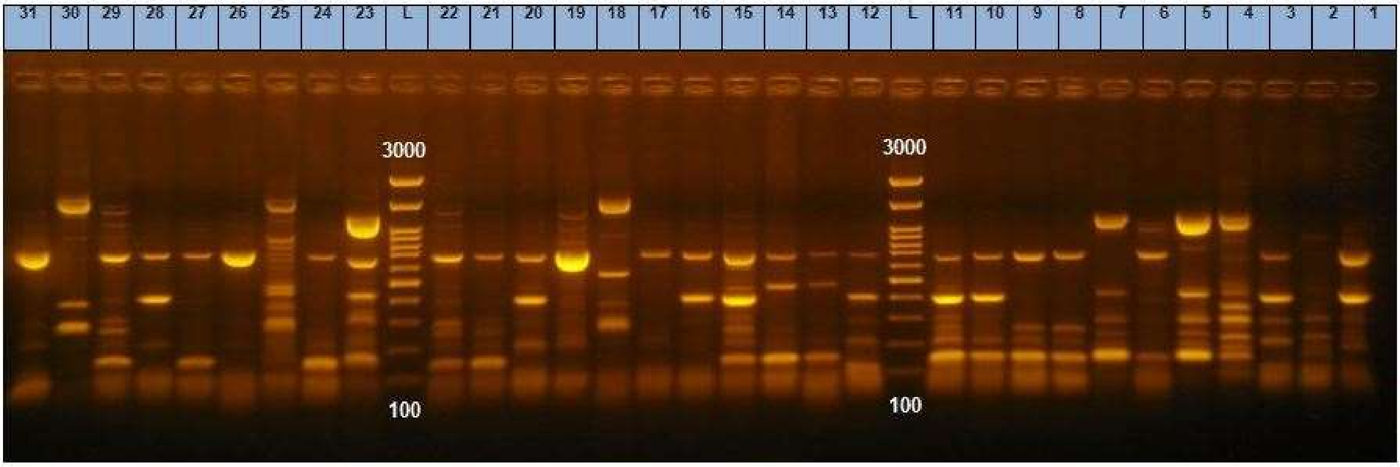

Figure S4. Agarose gel electrophoresis of ERIC fingerprints of 31 carbapenem-resistant Gram-negative bacterial isolates harboring plasmids on agarose gel electrophoresis; lane L, DNA size marker; lanes 1 through 31, numbered fingerprints. By visual inspection, samples 8 and 9 show close relatedness on the gel.

Table S1. Antibiogram analysis of carbapenem-resistant *Enterobacteriaceae* isolates (n=38)

	Resistance profile against																
**Isolate code**	**Imipenem**	**Meropenem**	**Ertapenem**	**Doripenem**	**Amoxicillin/Clavulanate**	**Ampicillin/Sulbactam**	**Cefoxitin**	**Ceftazidime**	**Ceftriaxone**	**Cefepime**	**Amikacin**	**Gentamicin**	**Ciprofloxacin**	**Levofloxacin**	**Trimethoprim/sulfamethoxazole**	**Nitrofurantoin**	**Fosfomycin**
***Klebsiella pneumoniae* (n=22)**																	
**18.SK**	R	R	R	I	R	R	R	I	R	R	R	R	R	R	R	R	S
**21.SK**	S	S	R	S	S	S	S	S	S	S	R	R	R	R	R	R	S
**23.SK**	I	S	R	S	S	R	I	R	R	SDD	R	R	R	R	R	R	S
**24.SK**	I	R	R	I	R	R	R	R	I	SDD	S	S	R	R	S	R	S
**26.AK**	R	R	S	S	S	R	R	R	R	SDD	S	S	S	S	S	R	S
**27.AK**	R	R	R	R	I	R	R	R	R	R	R	R	R	R	R	R	S
**31.AK**	S	S	R	S	S	R	R	R	R	R	R	R	R	R	R	R	S
**32.AK**	R	R	R	R	I	R	R	R	R	R	S	I	R	R	I	R	S
**33.AK**	R	R	R	I	S	R	R	R	R	R	R	R	R	R	R	R	S
**36.AK**	I	S	R	S	S	S	S	R	R	R	R	R	R	R	R	R	R
**37.AK**	R	R	R	S	S	R	R	R	R	R	R	R	R	R	R	S	R
**42.WK**	R	R	R	R	R	R	R	R	R	R	R	R	R	R	R	R	R
**44.WK**	R	R	R	R	R	R	R	R	R	R	R	S	R	R	R	R	S
**47.WK**	S	S	R	S	S	R	R	R	R	R	S	S	R	R	R	R	S
**53.WK**	R	R	R	I	R	R	R	R	R	R	R	R	R	R	R	R	S
**56.WK**	I	S	R	S	S	R	R	R	R	R	R	I	R	R	R	R	S
**57.WK**	R	R	R	R	I	R	R	R	R	R	R	S	R	R	I	R	S
**59.WK**	R	R	R	R	R	R	R	R	R	R	R	R	R	R	R	R	S
**60.WK**	R	R	R	R	R	R	R	R	R	R	R	R	R	R	R	R	S
**64.WK**	R	S	S	S	S	R	R	R	R	SDD	S	S	I	S	R	S	S
**66.WK**	I	R	R	R	I	R	R	R	R	R	R	S	R	R	R	R	S
**67.WK**	R	R	R	R	R	R	R	R	R	R	R	R	R	R	S	R	S
***Klebsiella terrigena* (n=6)**																	
**6.SK**	R	R	R	R	R	R	R	R	R	R	R	R	R	R	R	R	S
**11.SK**	R	R	R	S	R	R	R	R	R	R	I	S	R	R	S	R	S
**14.SK**	S	S	R	S	S	S	R	S	S	S	R	R	R	R	R	R	S
**28.AK**	I	R	R	R	R	R	R	R	R	R	R	R	R	R	R	R	S

	Resistance profile against																
**Isolate code**	**Imipenem**	**Meropenem**	**Ertapenem**	**Doripenem**	**Amoxicillin/Clavulanate**	**Ampicillin/Sulbactam**	**Cefoxitin**	**Ceftazidime**	**Ceftriaxone**	**Cefepime**	**Amikacin**	**Gentamicin**	**Ciprofloxacin**	**Levofloxacin**	**Trimethoprim/sulfamethoxazole**	**Nitrofurantoin**	**Fosfomycin**
**35.AK**	R	R	R	R	R	R	R	I	R	R	R	R	R	R	R	R	S
**41.WK**	R	R	R	R	R	R	R	R	R	R	R	R	R	R	R	R	S
***Escherichia coli* (n=6)**																	
**16.SE**	R	S	R	S	S	R	R	R	R	R	S	S	R	R	R	S	S
**20.SE**	S	S	R	S	S	I	R	R	R	R	S	S	R	R	R	S	S
**29.AE**	S	S	R	S	S	S	S	R	R	R	S	S	R	R	R	S	S
**30.AE**	I	S	R	S	R	R	R	R	R	R	S	S	R	R	R	S	S
**34.AE**	I	S	R	S	S	I	R	R	R	R	S	I	R	R	R	S	S
**48.WE**	R	R	R	R	S	R	R	R	R	R	S	S	R	R	R	S	S
***Proteus mirabilis* (n=2)**																	
**58.WPr**	R	R	R	R	R	R	R	R	R	R	R	R	R	R	R	S	S
**65.WPr**	I	S	R	S	S	I	S	S	S	S	S	S	R	I	R	S	I
***Enterobacter cloacae* (n=2)**																	
**3.SEn**	S	S	R	S	S	R	R	R	R	R	S	R	R	R	R	S	S
**25.SEn**	S	S	R	S	S	S	I	R	R	SDD	S	R	R	R	R	S	R

Abbreviations: S, sensitive; R; resistant; I, intermediate sensitivity; SDD, susceptible dose-dependent (a breakpoint category for which the susceptibility of an isolate depends on the dosing regimen used).

Table S2. Antibiogram analysis of carbapenem-resistant *Pseudomonas aeruginosa* isolates (n=19)

	**Resistance profile against**								
**Isolate code**	**Imipenem**	**Meropenem**	**Doripenem**	**Ceftazidime**	**Cefepime**	**Amikacin**	**Gentamicin**	**Ciprofloxacin**	**Levofloxacin**
**1.SP**	R	S	S	S	S	S	S	R	R
**2.SP**	R	R	S	R	I	I	R	R	R
**4.SP**	R	R	R	R	R	R	R	R	R
**5.SP**	R	R	R	R	R	I	R	R	R
**7.SP**	I	R	I	R	R	R	R	R	R
**8.SP**	R	R	S	R	R	S	R	R	R
**9.SP**	R	R	S	R	R	R	R	R	R
**10.SP**	R	R	S	R	R	S	R	R	R
**12.SP**	R	R	S	R	R	I	R	R	R
**13.SP**	R	R	S	R	R	S	R	R	R
**15.SP**	R	R	R	R	I	R	R	R	R
**19.SP**	R	R	S	R	R	S	S	R	R
**22.SP**	I	R	S	R	I	S	R	R	R
**39.WP**	R	R	R	R	R	R	R	R	R
**43.WP**	R	R	R	R	R	R	R	R	R
**50.WP**	R	R	R	R	R	R	R	R	R
**54.WP**	R	R	R	R	R	R	R	R	R
**61.WP**	R	R	R	R	R	R	R	R	R
**62.WP**	R	R	S	R	R	I	R	R	R

Abbreviations: S, sensitive; R; resistant; I, intermediate sensitivity.

Table S3. Antibiogram analysis of carbapenem-resistant *Acinetobacter baumannii* isolates (n=8)

	Resistance profile against											
**Isolate code**	**Imipenem**	**Meropenem**	**Doripenem**	**Ampicillin/Sulbactam**	**Ceftazidime**	**Ceftriaxone**	**Cefepime**	**Amikacin**	**Gentamicin**	**Ciprofloxacin**	**Levofloxacin**	**Trimethoprim/sulfamethoxazole**
**38.WA**	R	R	R	I	R	R	R	R	R	R	R	S
**40.WA**	R	S	S	R	R	R	R	R	R	R	I	R
**45.WA**	R	R	S	S	R	R	R	R	R	R	R	S
**46.WA**	R	R	I	S	R	R	R	R	R	R	R	I
**49.WA**	R	R	S	R	R	R	R	R	R	R	S	R
**51.WA**	R	R	S	S	S	S	I	R	R	R	S	R
**52.WA**	R	R	R	I	R	R	R	R	R	R	R	S
**55.WA**	R	R	I	S	R	R	R	R	R	R	R	R

Abbreviations: S, sensitive; R; resistant; I, intermediate sensitivity.
